# Complete Closed Genome Sequences of Four *Mannheimia varigena* Isolates from Cattle with Shipping Fever

**DOI:** 10.1128/genomeA.00088-14

**Published:** 2014-02-13

**Authors:** Gregory P. Harhay, Robert W. Murray, Brian Lubbers, Dee Griffin, Sergey Koren, Adam M. Phillippy, Dayna M. Harhay, James Bono, Michael L. Clawson, Michael P. Heaton, Carol G. Chitko-McKown, Timothy P. L. Smith

**Affiliations:** a U.S. Department of Agriculture, ARS, U.S. Meat Animal Research Center (USMARC), Clay Center, Nebraska, USA; b Zoetis Inc., Kalamazoo, Michigan, USA; c Kansas State University College of Veterinary Medicine, Veterinary Diagnostic Laboratory, Manhattan, Kansas, USA; d University of Nebraska, Great Plains Veterinary Educational Center, Clay Center, Nebraska, USA; e National Biodefense Analysis and Countermeasures Center, Frederick, Maryland, USA

## Abstract

*Mannheimia varigena* is an occasional respiratory pathogen of cattle and pigs. We present the first four complete closed genome sequences of this species.

## GENOME ANNOUNCEMENT

*Mannheimia varigena* is a Gram-negative rod bacterium that is infrequently associated with severe acute hemorrhagic fibrinonecrotic bronchopneumonia in feedlot cattle (1). *M. varigena* is an opportunistic pathogen that can be found in the nasopharynxes of asymptomatic cattle and other ruminants (2, 3). The bovine respiratory disease complex (BRDC) progression to bronchopneumonia results from host, pathogen, and environmental interactions that are not completely understood. Stress, viral infections, extreme weather changes, transportation, and other factors appear to predispose cattle to bronchopneumonia that is symptomatic of BRDC (4). Little is known of the genetic elements that may enhance or contribute to the virulence potential of *M. varigena*. Accordingly, we report here the first four complete closed genome sequences of *M. varigena*. All sequenced isolates were recovered from lung tissue samples at necropsy from animals with shipping fever.

Species identification of *M. varigena* isolates was verified on the OmniLog microbial identification system (Biolog, Hayward, CA). Genomic DNA of *M. varigena* isolates USDA-ARS-USMARC-1261, -1296, -1312, and -1388 was extracted using a blood and cell culture DNA kit (Qiagen, Valencia CA). Sequencing was performed on a Pacific Biosciences RSII instrument (Pacific Biosciences, Menlo Park, CA) using libraries prepared with the manufacturer’s kits at a 15-kb insert size and with the P4/C2 version of chemistry. The read coverages for the four genomes varied from 11- to 25-fold after error correction with PBcR (5), while the minimum read length varied from 6 to 11 kb. Reads were assembled using Celera assembler version 7 (5), which produced a single large contig for each isolate that was then validated and improved using Quiver (6). For all isolates, a self-self dot plot of the consensus sequences revealed at least a 1.5-kb overlap between the ends of the contig at >99% identity, consistent with a circular chromosome. Duplicated sequence was removed from the 3′ end of each isolate to generate the proper circularized sequence. The origin of replication was approximated using GenSkew (http://genskew.csb.univie.ac.at), and a new linear model of the chromosome was generated using this origin position as base 1. The validity of the circularization was verified by mapping all the raw PacBio reads to this final model (including across the junction where circularization had been enforced) using Quiver, which also resolves remaining sequence errors to generate assemblies with >99.9% accuracy. A local instance of Do-It-Yourself Annotator (DIYA) (7) was used to annotate the circularized chromosome.

For *M. varigena* USDA-ARS-USMARC-1261, -1296, -1312, and -1388, the genome sizes were determined to be 2,393,449, 2,245,238, 2,131,927, and 2,228,291 bp, with gene counts of 2,622, 2,190, 2,062, and 2,176, coding sequence (CDS) counts of 2,381, 2,109, 1,982, and 2,096, and tRNA counts of 61, 61, 60, and 60, respectively, and rRNA counts of 20.

### Nucleotide sequence accession numbers.

The GenBank nucleotide sequence accession numbers for USDA-ARS-USMARC-1261, -1296, -1312, and -1388 are CP006942, CP006943, CP006944, and CP006953, respectively.
